# Supplementary material for: Morindone from Morinda citrifolia as a potential antiproliferative agent against colorectal cancer cell lines
Source: PLoS One. 2022 Jul 12;17(7):e0270970. doi: 10.1371/journal.pone.0270970 (PMC9275698; doi:10.1371/journal.pone.0270970)
Supplement: S1 File — (PDF) [file pone.0270970.s001.pdf]

S1 Fig. GCMS and 1D-NMR spectrum of Nordamnacanthal (1).

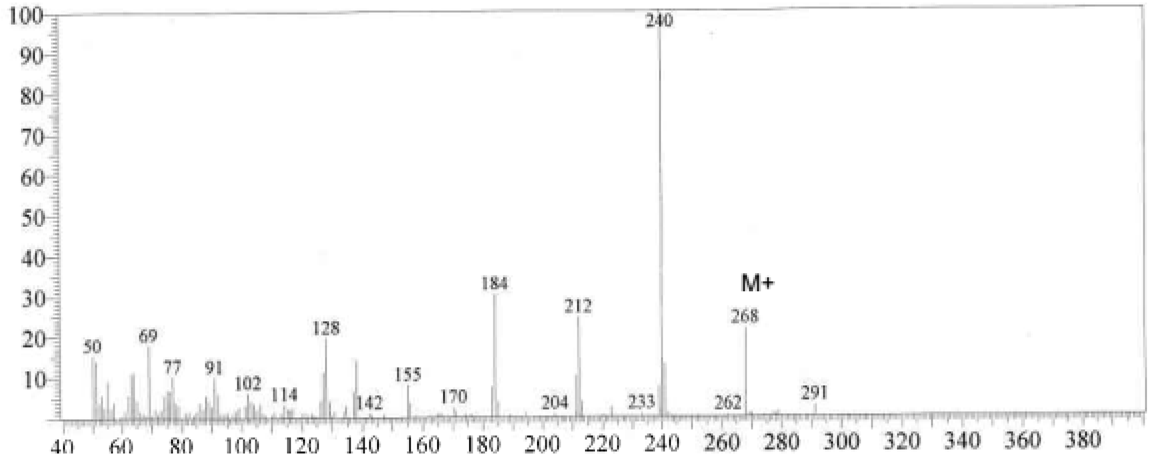

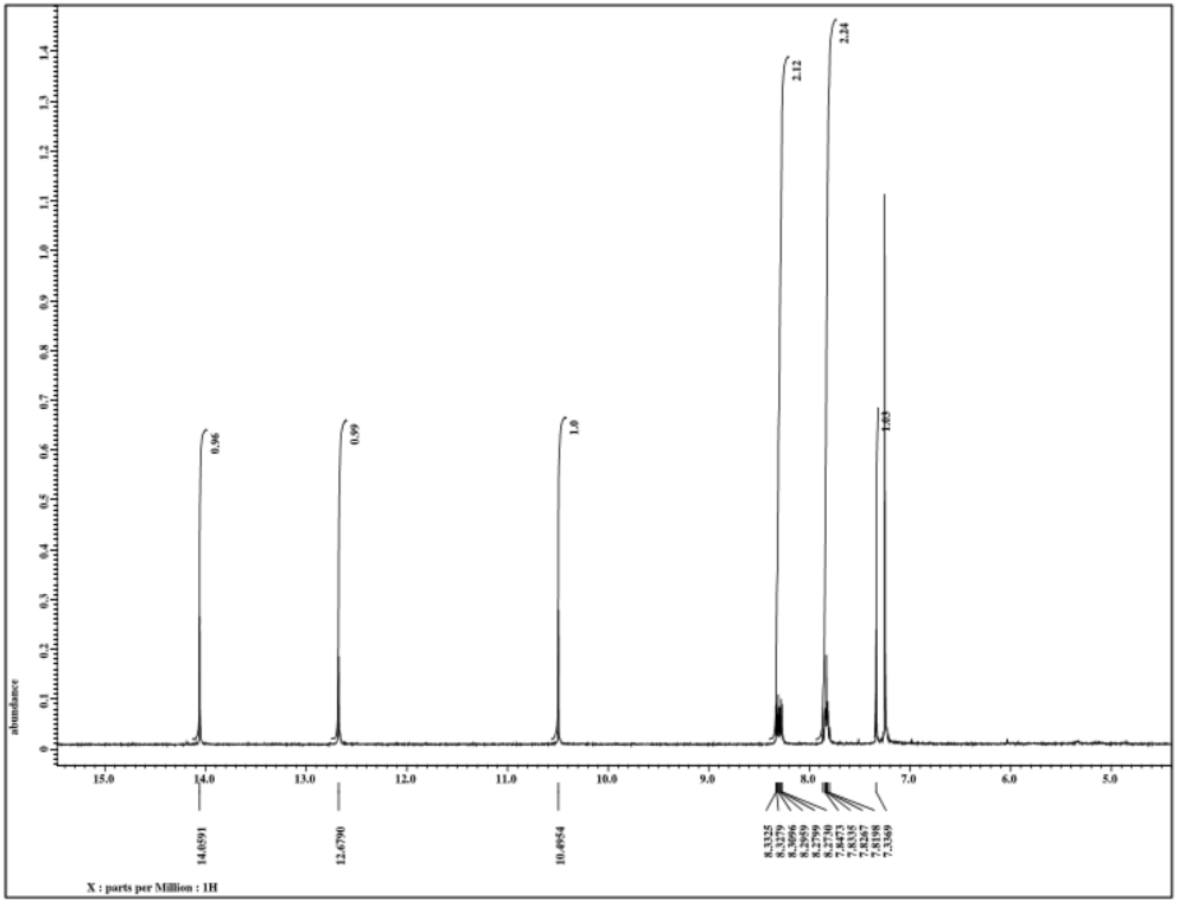
abundance
0
0.1
0.2
0.3
0.4
0.5
0.6
0.7
0.8
0.9
1.0
1.1
1.2
1.3
1.4
0.96
0.99
1.0
2.12
2.24
1.03
15.0
14.0
13.0
12.0
11.0
10.0
9.0
8.0
7.0
6.0
5.0
14.0591
12.6790
10.4954
8.3325
8.3279
8.3096
8.2959
8.2799
8.2730
7.8473
7.8335
7.8267
7.8198
7.3369
X : parts per Million : 1H

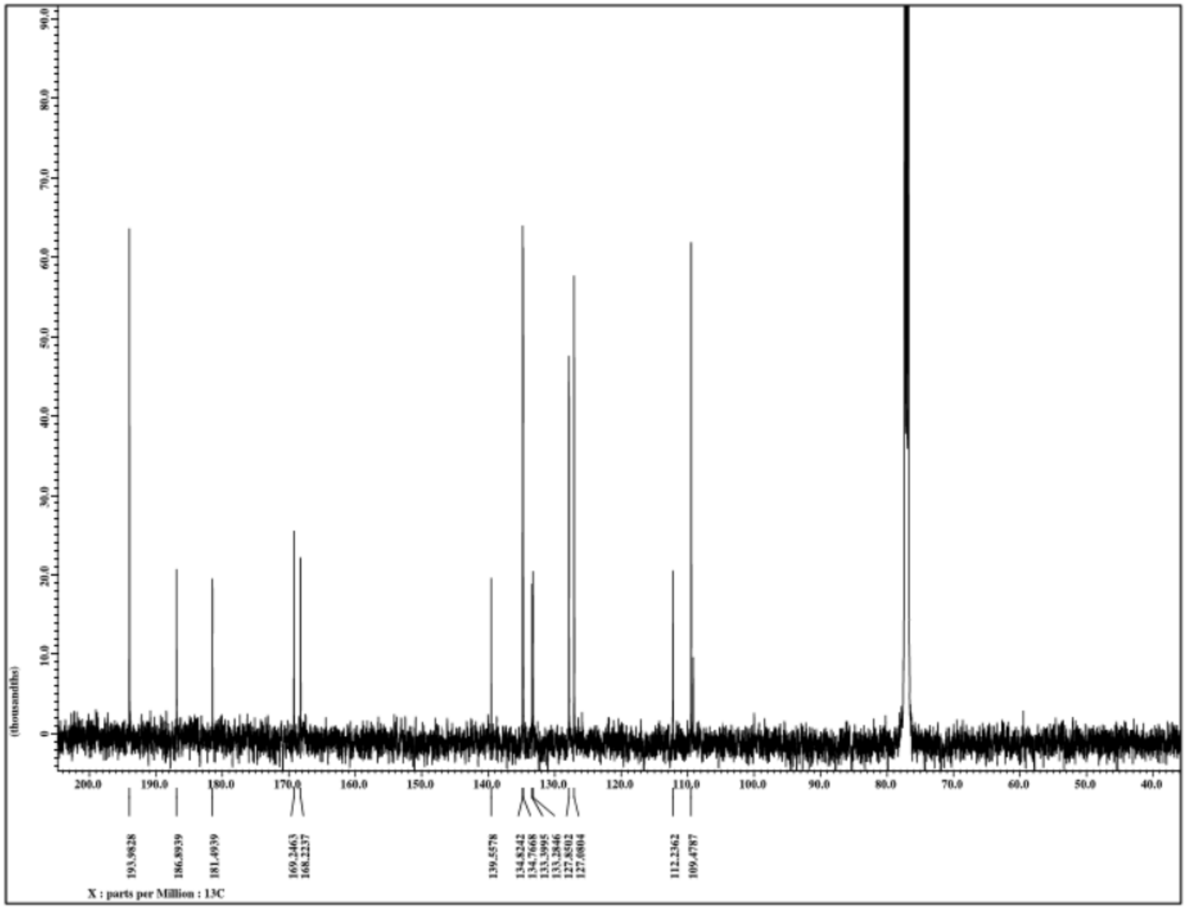
(thousandths)
193.9828
186.8939
181.4939
169.2463
168.2237
139.5578
134.8242
134.7668
133.3995
133.2846
127.8502
127.0804
112.2362
109.4787
X : parts per Million : 13C

S2 Fig. GCMS and 1D-NMR spectrum of Damnacanthal (2).

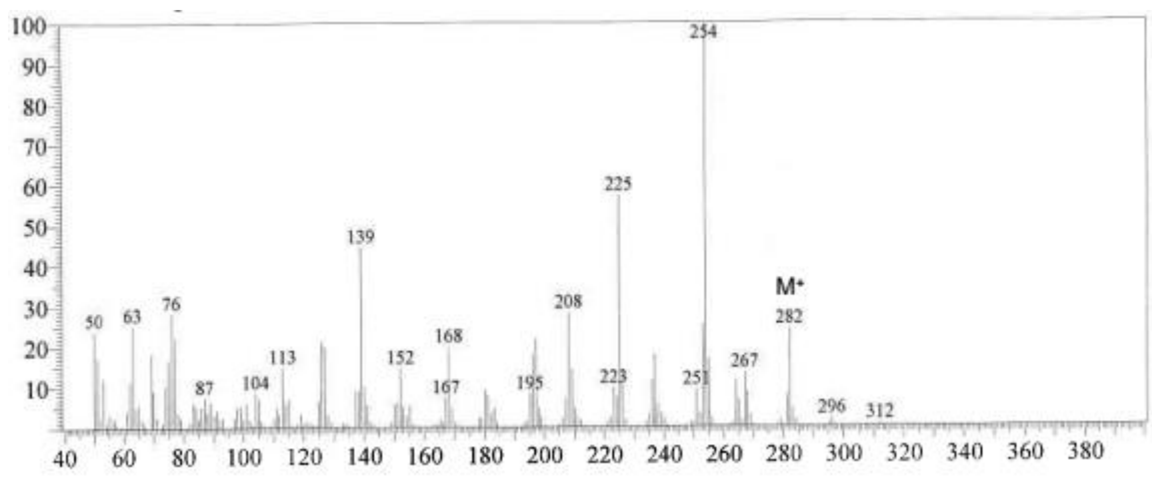

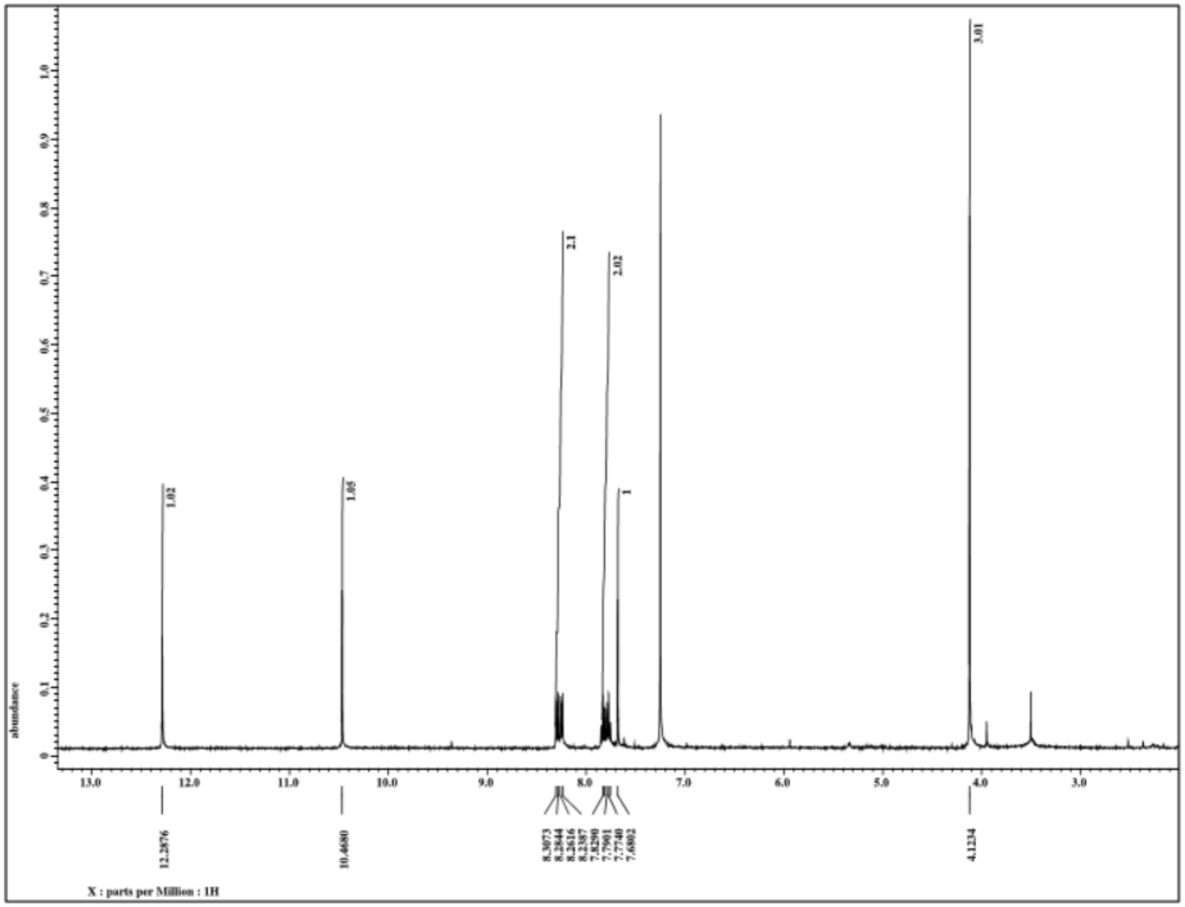

abundance
X : parts per Million : 1H
12.2876
10.4680
8.3073
8.2844
8.2616
8.2387
7.8290
7.7901
7.7740
7.6802
4.1234
1.02
1.05
2.1
2.02
1
3.01

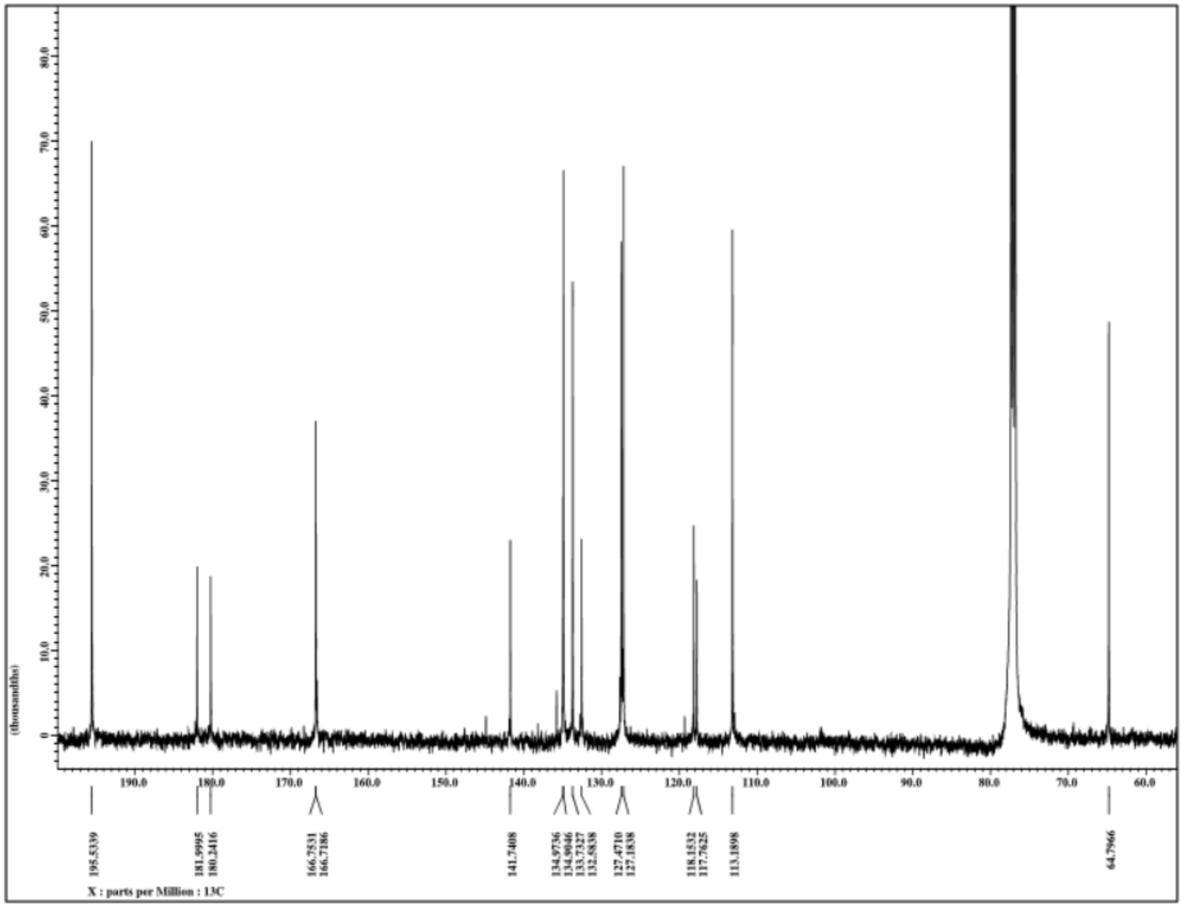
(thousandths)
80.0
70.0
60.0
50.0
40.0
30.0
20.0
10.0
0
190.0
180.0
170.0
160.0
150.0
140.0
130.0
120.0
110.0
100.0
90.0
80.0
70.0
60.0
195.5339
181.9995
180.2416
166.7531
166.7186
141.7408
134.9736
134.9046
133.7327
132.5838
127.4710
127.1838
118.1532
117.7625
113.1898
64.7966
X : parts per Million : 13C

S3 Fig. GCMS and 1D-NMR spectrum of 1,3,5-trihydroxy-2-methoxy-6-methyl anthraquinone (3).

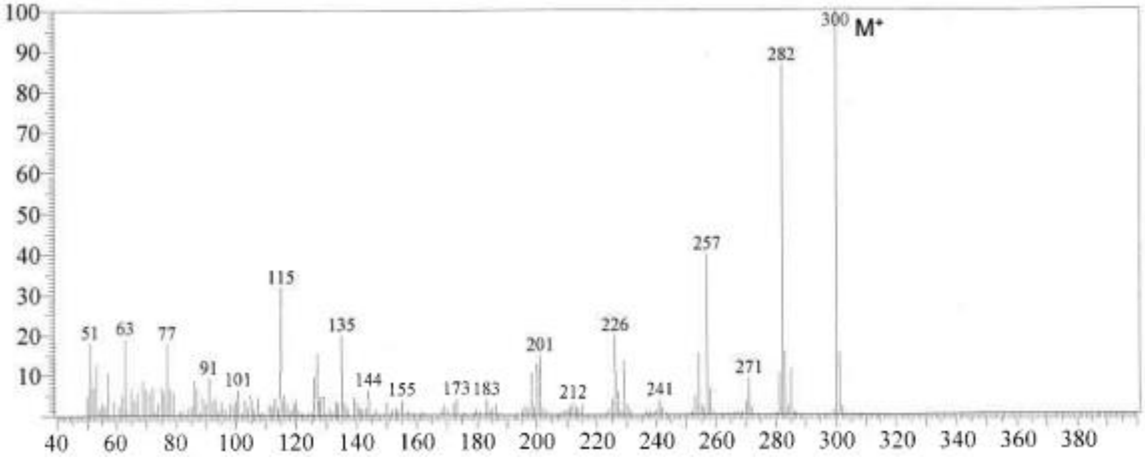

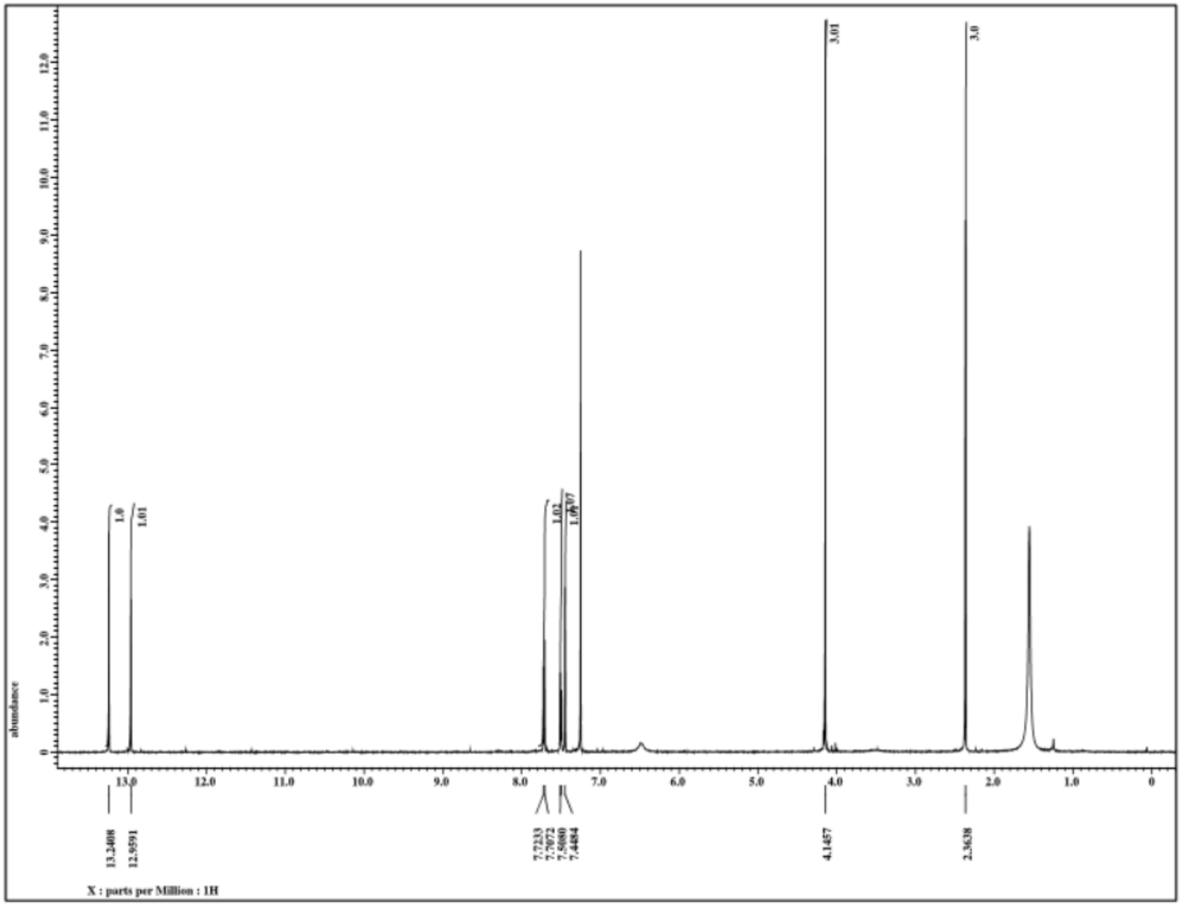

abundance
13.2408
12.9591
7.7233
7.7072
7.5080
7.4484
4.1457
2.3638
X : parts per Million : 1H

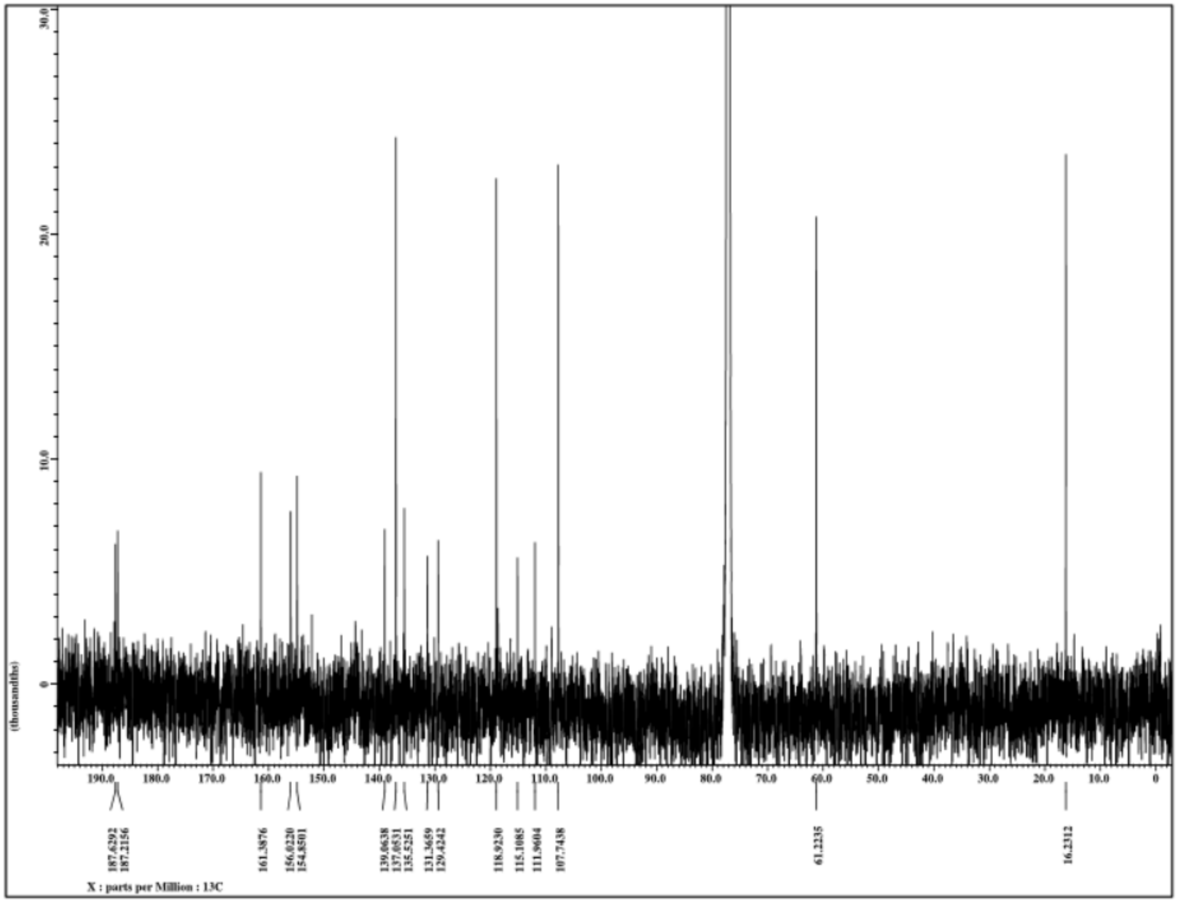

(thousandths)
30.0
20.0
10.0
0
190.0 180.0 170.0 160.0 150.0 140.0 130.0 120.0 110.0 100.0 90.0 80.0 70.0 60.0 50.0 40.0 30.0 20.0 10.0 0
187.6292
187.2156
161.3876
156.0220
154.8501
139.0638
137.0531
135.5251
131.3659
129.4242
118.9230
115.1065
111.9604
107.7438
61.2235
16.2312
X : parts per Million : 13C

S4 Fig. GCMS and 1D-NMR spectrum of Sorendidiol (5).

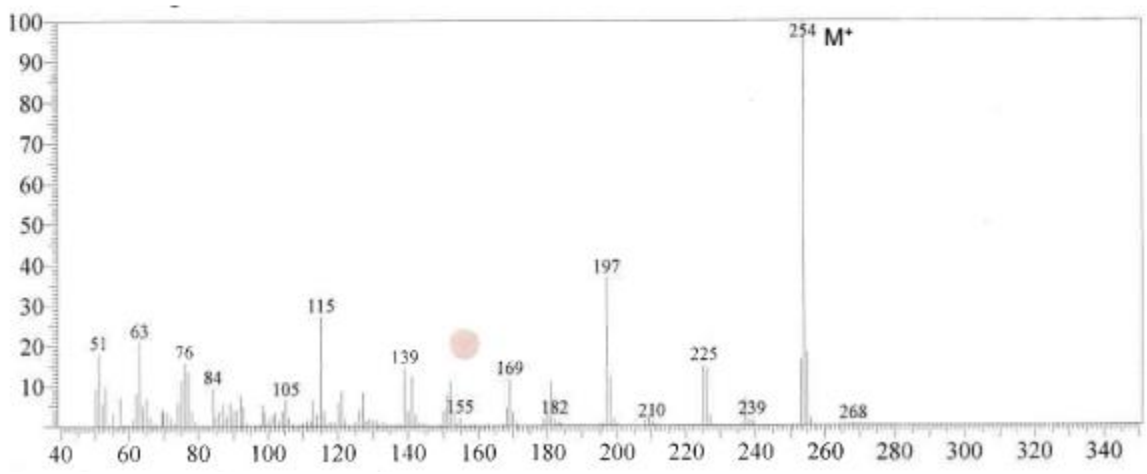

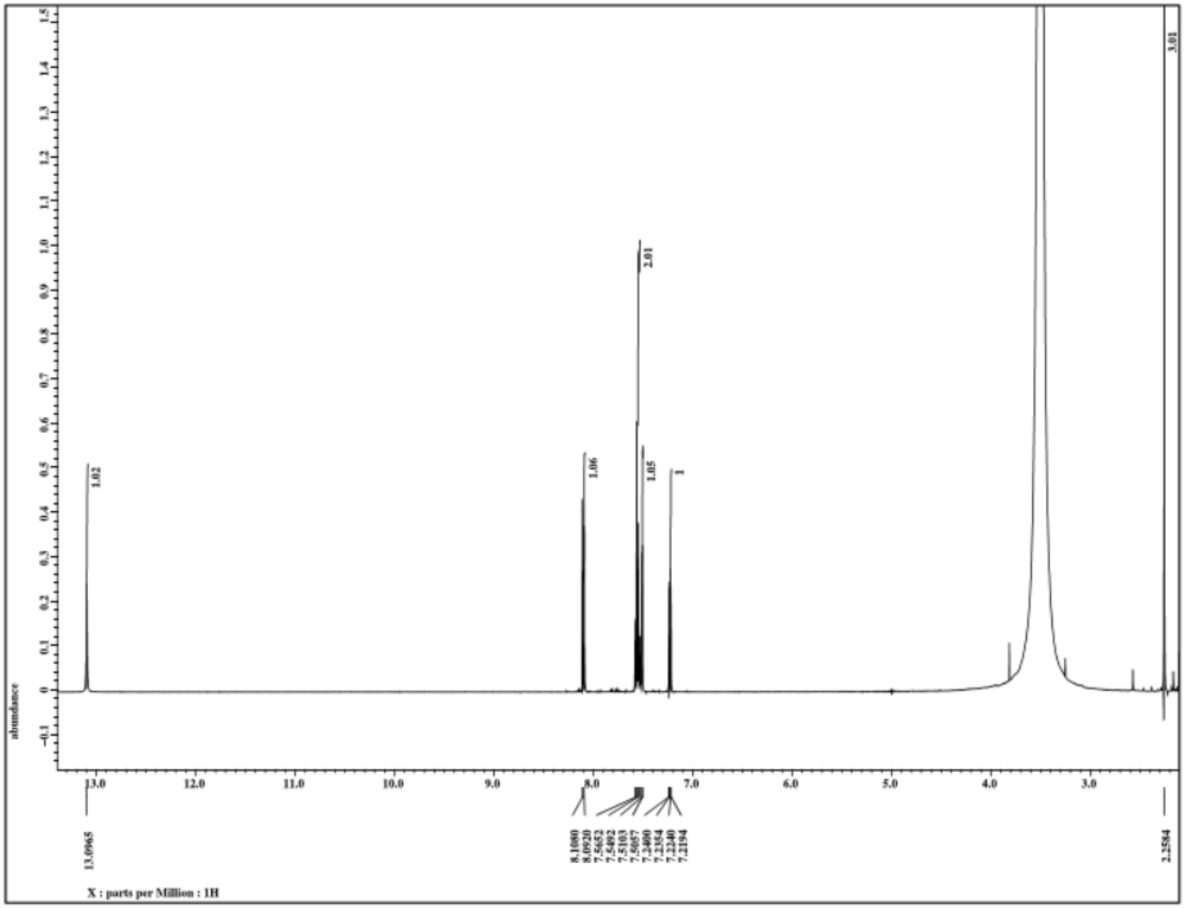

abundance
1.5
1.4
1.3
1.2
1.1
1.0
0.9
0.8
0.7
0.6
0.5
0.4
0.3
0.2
0.1
0
-0.1
1.02
1.06
2.01
1.05
1
3.01
13.0
12.0
11.0
10.0
9.0
8.0
7.0
6.0
5.0
4.0
3.0
13.0965
8.1080
8.0920
7.5652
7.5492
7.5103
7.5057
7.2400
7.2354
7.2340
7.2194
2.2584
X : parts per Million : 1H

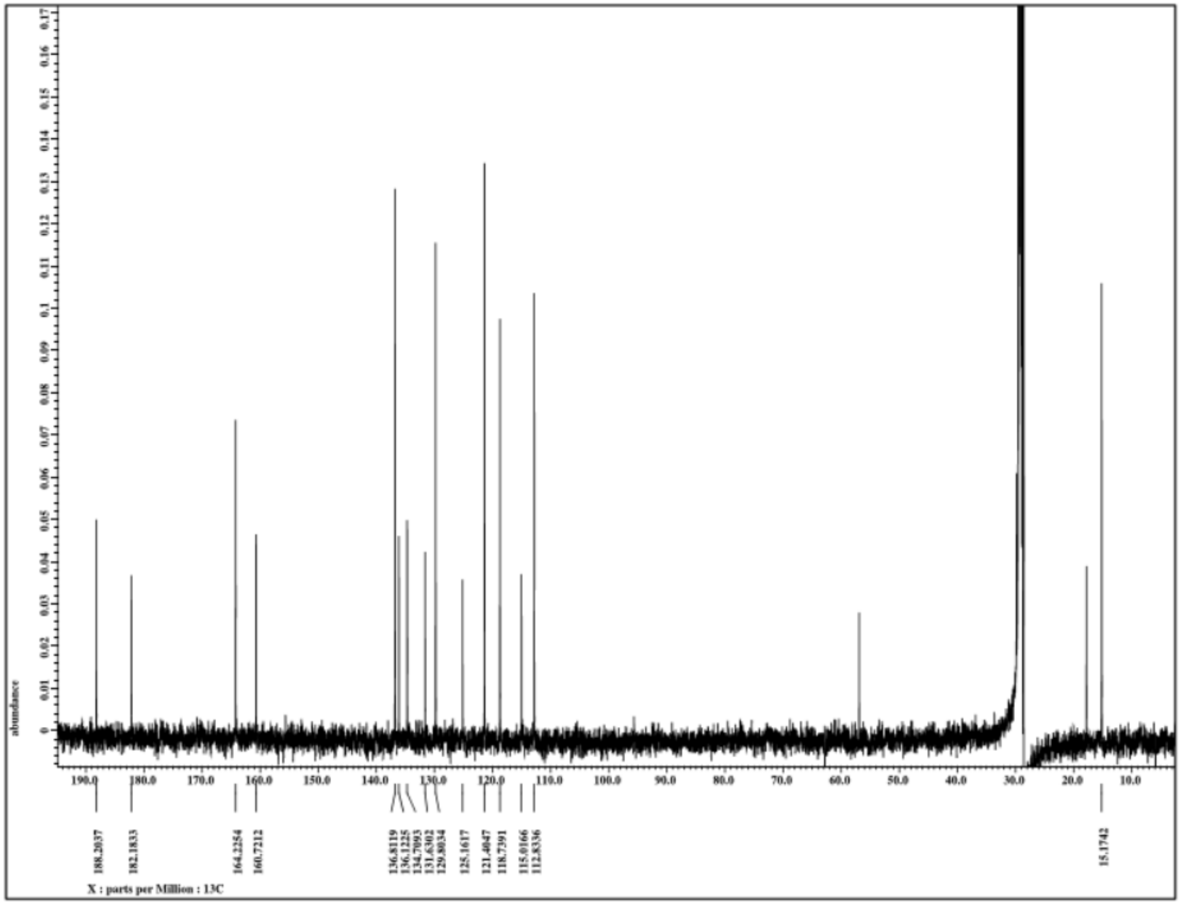

abundance
X : parts per Million : 13C
188.2037
182.1833
164.2254
160.7212
136.8119
136.1225
134.7093
131.6302
129.8034
125.1617
121.4047
118.7391
115.0166
112.8336
15.1742

S5 Fig. GCMS and 1D-NMR spectrum of Rubiadin (6).

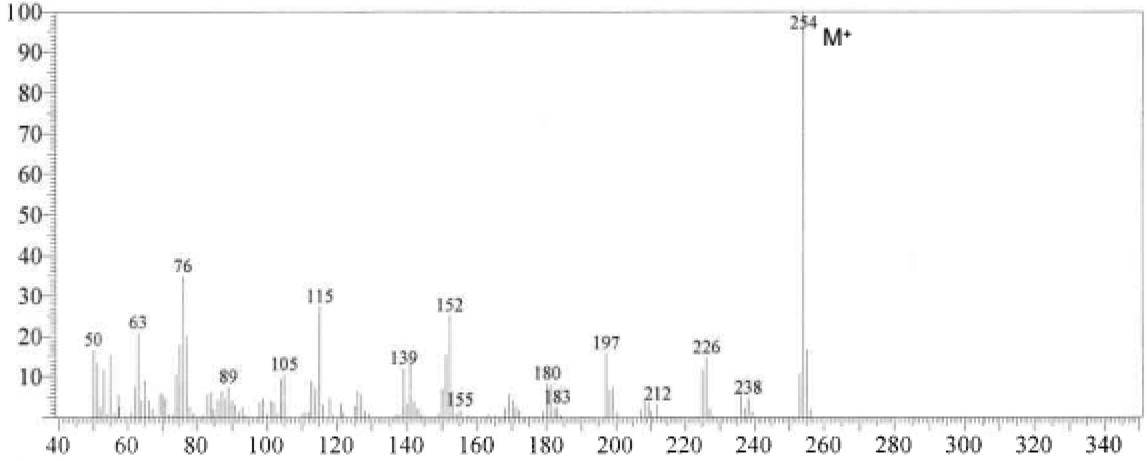

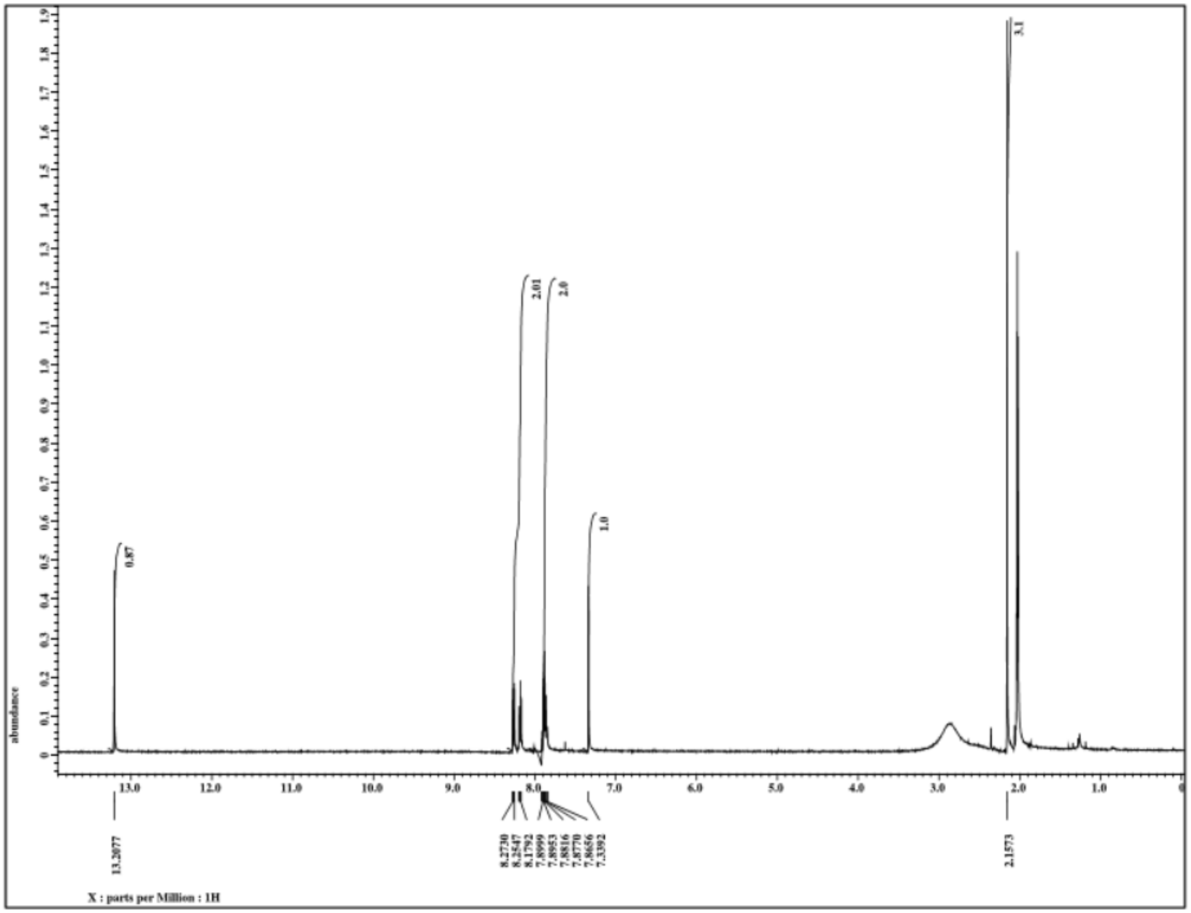

abundance
0.87
2.01
2.0
1.0
3.1
13.2077
8.2730
8.2547
8.1792
7.8999
7.8953
7.8816
7.8770
7.8656
7.3392
2.1573
X : parts per Million : 1H

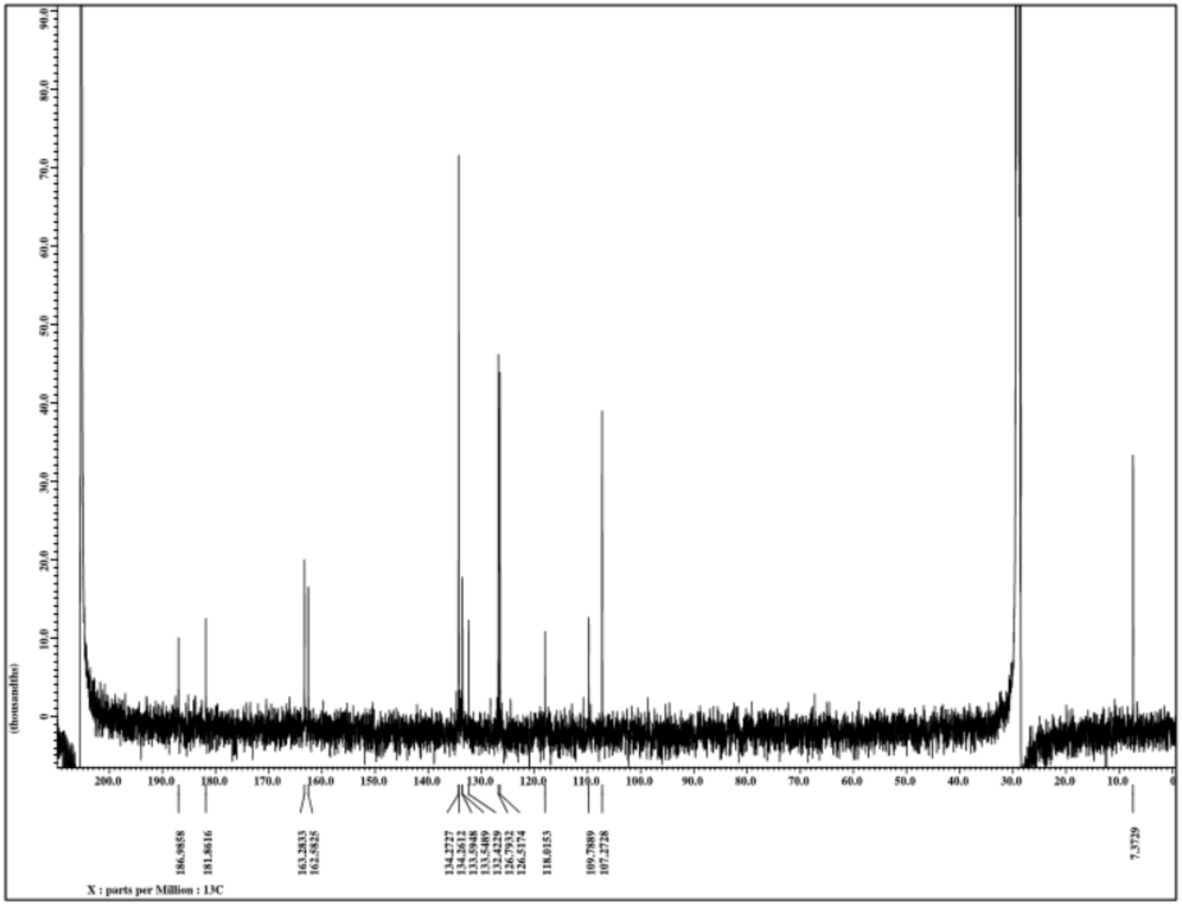

(thousandths)
90.0
80.0
70.0
60.0
50.0
40.0
30.0
20.0
10.0
0
200.0
190.0
180.0
170.0
160.0
150.0
140.0
130.0
120.0
110.0
100.0
90.0
80.0
70.0
60.0
50.0
40.0
30.0
20.0
10.0
0
186.9858
181.8616
163.2833
162.5825
134.2727
134.2612
133.5948
133.5489
132.4229
126.7932
126.5174
118.0153
109.7889
107.2728
7.3729
X : parts per Million : 13C

S6 Fig. GCMS and 1D-NMR spectrum of Damnacanthol (7).

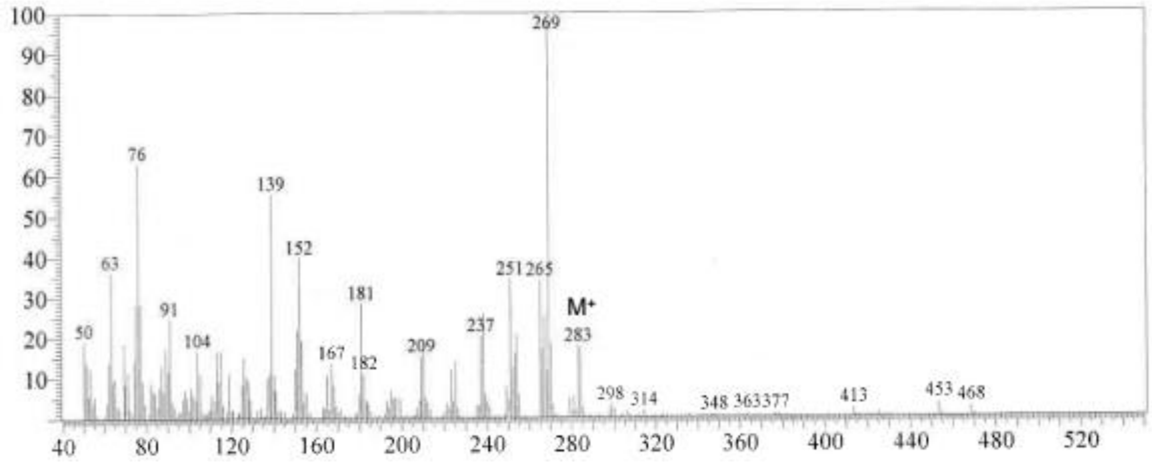

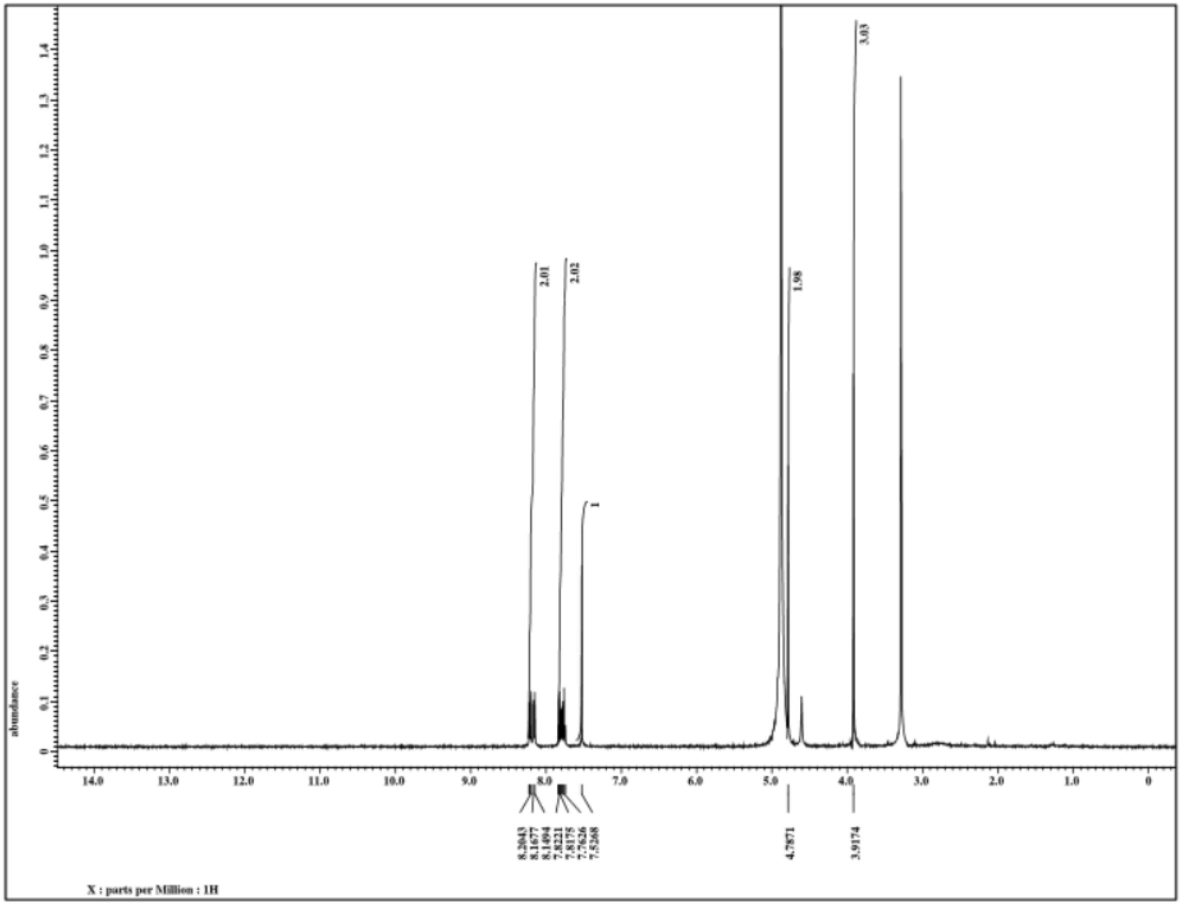

abundance
2.01
2.02
1
1.98
3.03
8.2043
8.1677
8.1494
7.8221
7.8175
7.7626
7.5268
4.7871
3.9174
X : parts per Million : 1H

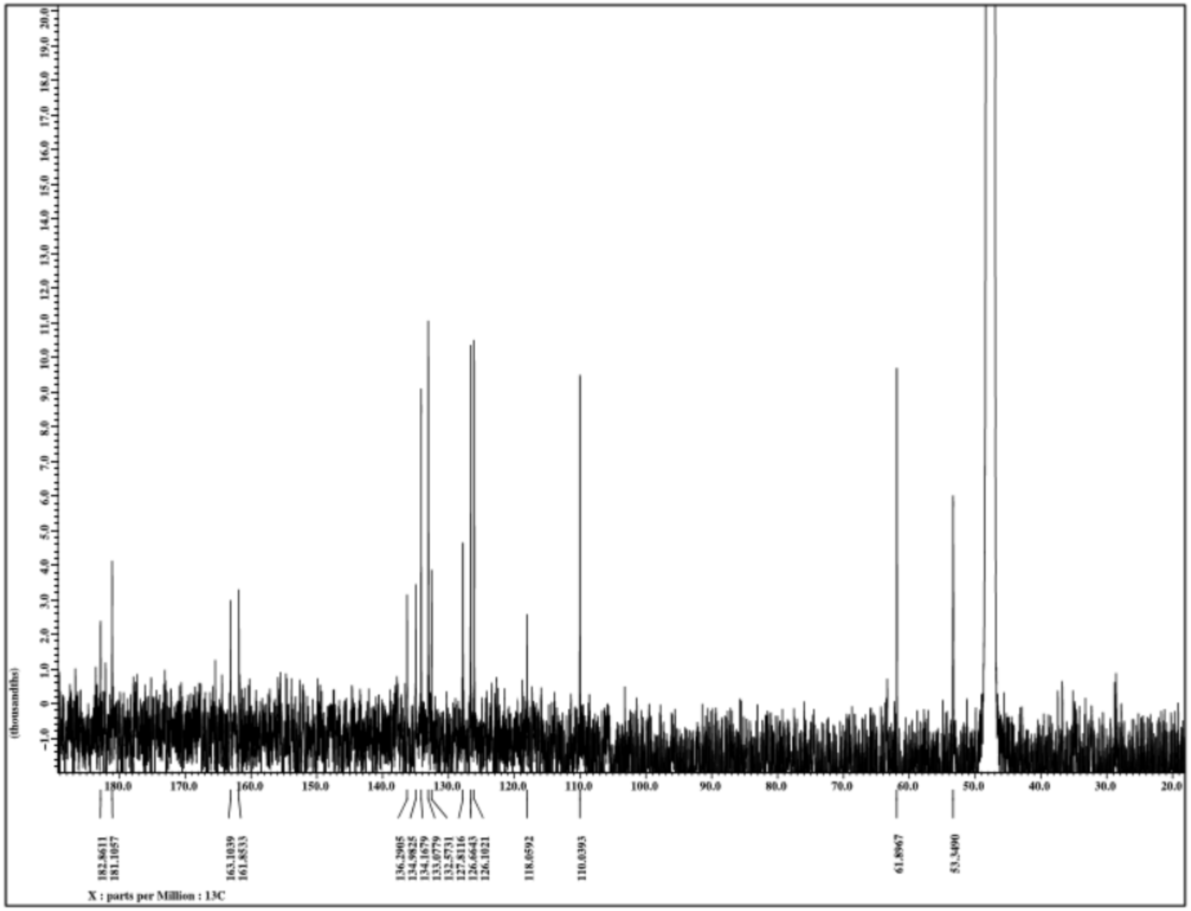

(thousandths)
182.8611
181.1057
163.1039
161.8533
136.2905
134.9825
134.1679
133.0779
132.5731
127.8116
126.6643
126.1021
118.0592
110.0393
61.8967
53.3490
X : parts per Million : 13C

S7 Fig. GCMS and 1D-NMR spectrum of Lucidin-ω-methylether (8).

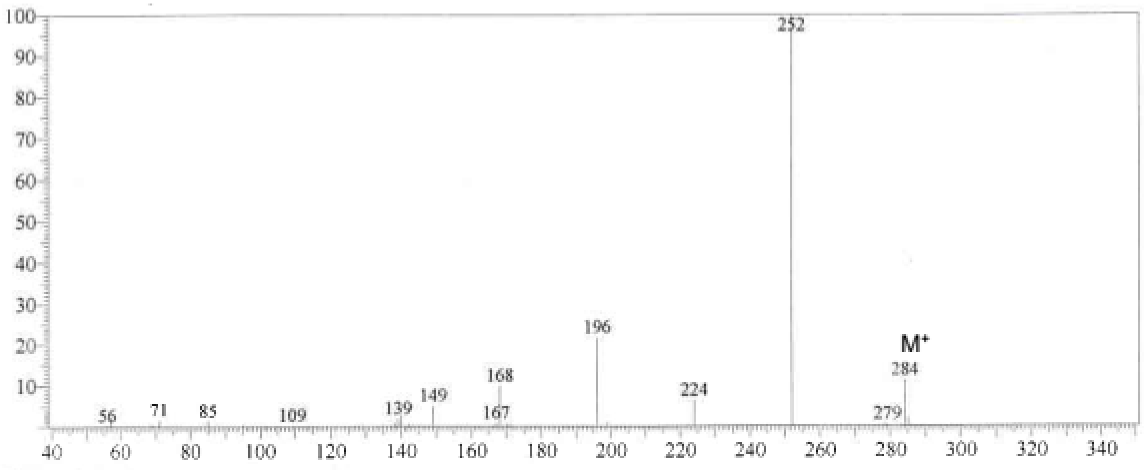

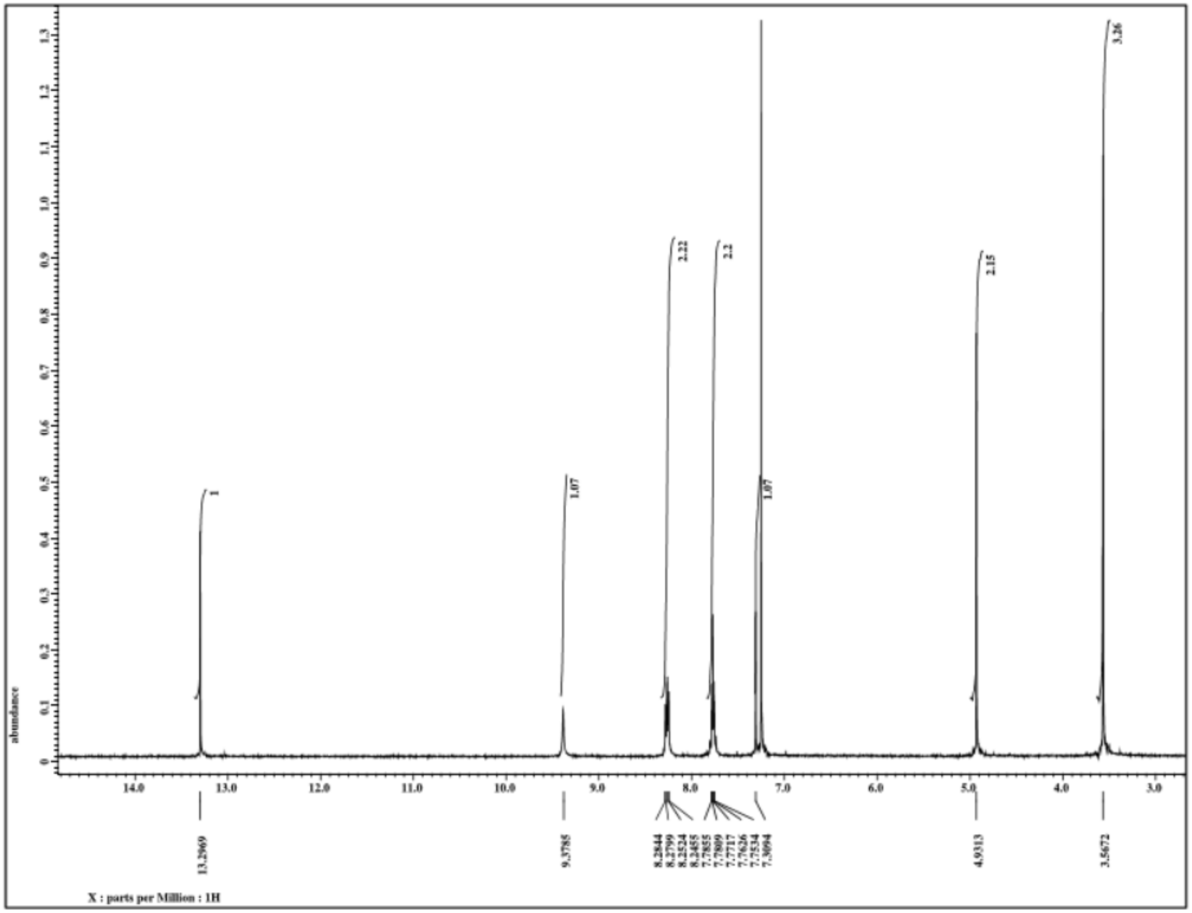
abundance
X : parts per Million : 1H
13.2969
9.3785
8.2844
8.2799
8.2524
8.2455
7.7855
7.7809
7.7717
7.7626
7.7534
7.3094
4.9313
3.5672
1
1.07
2.22
2.2
1.07
2.15
3.26

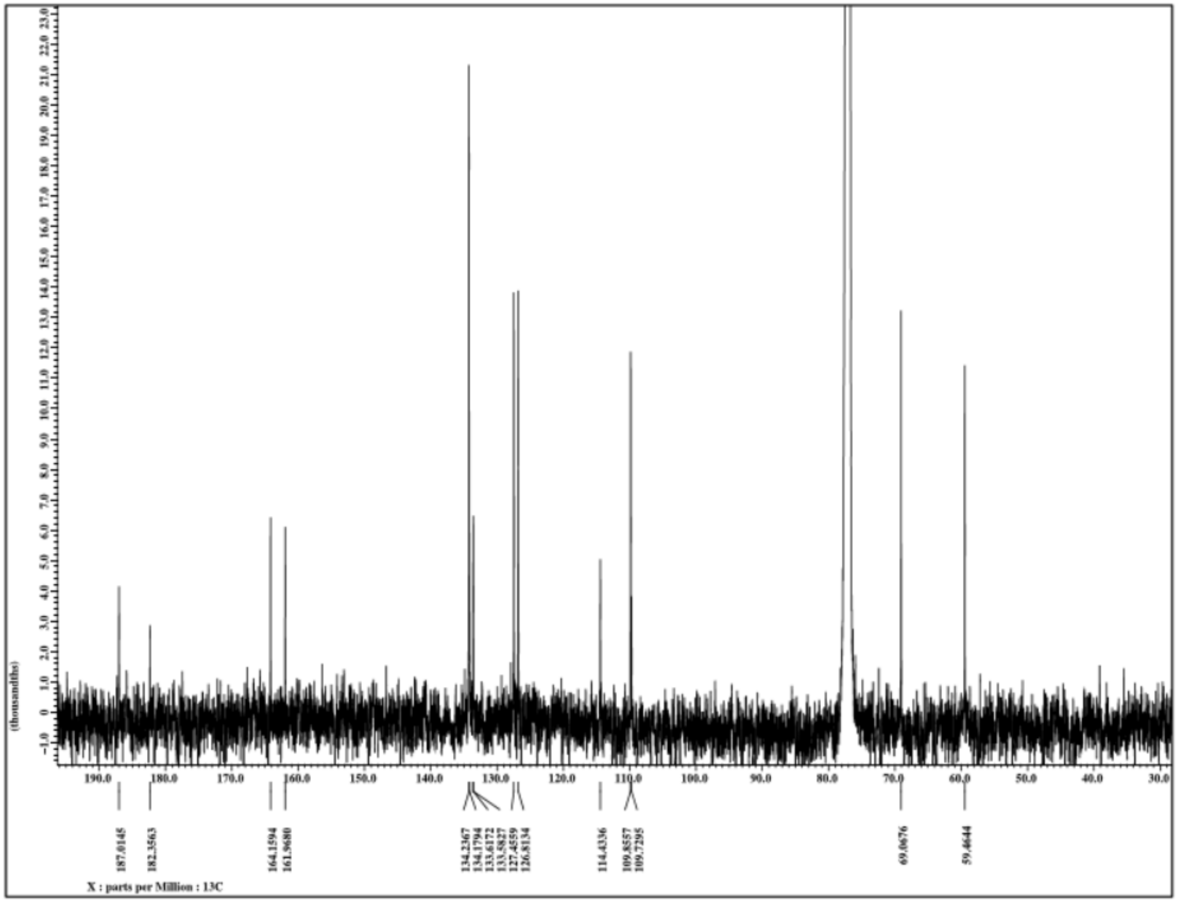
(thousandths)
X : parts per Million : 13C
187.0145
182.3563
164.1594
161.9680
134.2367
134.1794
133.6172
133.5827
127.4559
126.8134
114.4336
109.8557
109.7295
69.0676
59.4644
