# Supplementary material for: Morindone from Morinda citrifolia as a potential antiproliferative agent against colorectal cancer cell lines
Source: PLoS One. 2022 Jul 12;17(7):e0270970. doi: 10.1371/journal.pone.0270970 (PMC9275698; doi:10.1371/journal.pone.0270970)
Supplement: S1 Table — δ in ppm, J in Hz. (PDF) [file pone.0270970.s002.pdf]

S8 Table. ^{1}H NMR (400 MHz) and ^{13}C NMR (125 MHz) spectral data of morindone (4) in Acetone-d_6.

Position	Morindone (4) in Acetone-d_6	
	^{1}H NMR	^{13}C NMR
1	7.72 (d,1H, *J*=8.1)	118.6
2	7.64 (d,1H, *J*=8.1)	136.8
3	2.32 (s, 3H)	135.6
4	-	161.1
4a	-	115.3
5	7.78 (d,1H, *J*=8.0)	121.3
5a	-	124.3
6	7.27 (d,1H, *J*=8.0)	120.7
7	-	151.1
8	-	153.2
8a	-	116.4
9	-	188.6
9a	-	131.3
10	-	187.5
CH_3	-	14.2
4-OH	13.25 (s,1H)	-
8-OH	12.84 (s,1H)	-

δ in ppm, *J* in Hz.
