# Supplementary material for: Morindone from Morinda citrifolia as a potential antiproliferative agent against colorectal cancer cell lines
Source: PLoS One. 2022 Jul 12;17(7):e0270970. doi: 10.1371/journal.pone.0270970 (PMC9275698; doi:10.1371/journal.pone.0270970)
Supplement: Attachment — Submitted filename: Plos one review comments.docx [file pone.0270970.s003.docx]

**Review comments**

Manuscript Number: PONE-D-21-32928

Dr. Nurshamimi and coworkers describes the Morindone from *Morinda citrifolia* as a potential antiproliferative agent against colorectal cancer cell lines.

The work carried out by this group is extension work of Kamiya group but this group used three colorectal cancer cell lines and activity shown by all the three cell lines was good.

After isolation and *in vitro* studies, binding efficiency of all the isolated derivatives were investigated. From the studies it is confirmed that Morindone is showed moderate inhibitory activity against multitargets like β-catenin, MDM2-p53 and KRAS.

1) Provided IR stretching frequencies isolated anthraquinone derivatives is not clear. All the derivatives with hydroxyl group but authors not mentioned the stretching frequencies of in data.

2) Include ^1^H NMR and ^13^C NMR data of active molecule Morindone in the manuscript.

3) Include *in vivo* assay results.

After all these modification paper will be considered.

I am suggesting major revision.
