# Supplementary material for: Morindone from Morinda citrifolia as a potential antiproliferative agent against colorectal cancer cell lines
Source: PLoS One. 2022 Jul 12;17(7):e0270970. doi: 10.1371/journal.pone.0270970 (PMC9275698; doi:10.1371/journal.pone.0270970)
Supplement: Attachment — Submitted filename: Response to reviewers.docx [file pone.0270970.s004.docx]

**Response to reviewers**

**Title: *Morindone from morinda citrifolia as a potential antiproliferative agent against colorectal cancer cell lines***

**Reference number: PONE-D-21-32928**

**Reviewer #1:** Search for new phytochemicals, with anti-cancer properties is an ever green area of research in the field of drug discovery, In view of this the authors in the present manuscript entitled “Morindone from Morinda citrifolia as a potential antiproliferative agent against colorectal cancer cell lines” have studied the Anti-cancer potentials of root barks of Morinda citrifolia and isolated the eight anthraquinone derivatives.

Even though the anticancer properties of anthraquinone compounds from Morinda citrifolia is previously studied in this present work, the authors have identified the key targets of these active compounds by protein docking studies. They have identified the binding sites for these compounds in the three important genes including P53, KRAS and β- catenin which were routinely mutated in colorecral cancer (CRC).

Comment #1.

In the Cytotoxic assay carried out the authors have used three human colorectal cancer cell lines: HT29 (with p53 and APC mutation), LS174T (with KRAS mutation) and HCT116 (with KRAS mutation). In this study HCT116 wells were seeded at 7500 cells/well while other cells were seeded at 30,000 cells/well. What is the reason for varying the number of cells seeded in the other cell lines?

***Response: The cell seeding density for each cell line has been optimized according to their proliferation rate and confluency at the end of treatment, hence the differences. HCT 116 cells showed faster proliferation rate while the other cells showed slower proliferation rate.***

Comment #2.

In the Introduction part in the last paragraph one of the reference needs to be corrected. It is written as (et al., 2016), without the author’s name. mention the author’s name as well.

***Response: Thank you for the comment. This has been updated with Vancouver style referencing, which is the PloS One reference style. The changes can be tracked in the Introduction section in the revised manuscript.***

Comment #3.

In the figures 3,4,5,6 representing the Cytotoxicity effect of anthraquinone compounds 1-8 in different cancer cell lines and normal cells, the graph could be represented in a colored line, which makes the lines distinguishable clearly.

***Response: Thank you for the suggestion. We have changed them to the colored line graph in Figures 4 – 7.***

The authors have identified that Morindone has high cytotoxicity effect and great selectivity index towards colorectal cancer cell lines as well as strong binding affinities in in-vitro and in-silico investigation towards multiple protein targets of β-catenin, MDM2-p53, and KRAS in comparison to other anthraquinones. In detail Molecular docking studies have shown that all the active compounds can bind well into each targeted protein receptor by binding to important residues in the protein, explaining their inhibitory activities. Further in vivo assays with Morindone are needed for the development of potential lead compound against colorectal cancer cells.

Overall, the manuscript could be accepted for publication once the above comments are considered and thoroughly revised again for any topographical error.

**Reviewer #2:** Review comments

Manuscript Number: PONE-D-21-32928

Dr. Nurshamimi and coworkers describes the Morindone from Morinda citrifolia as a potential antiproliferative agent against colorectal cancer cell lines.

The work carried out by this group is extension work of Kamiya group but this group used three colorectal cancer cell lines and activity shown by all the three cell lines was good.

After isolation and in vitro studies, binding efficiency of all the isolated derivatives were investigated. From the studies it is confirmed that Morindone is showed moderate inhibitory activity against multitargets like β-catenin, MDM2-p53 and KRAS.

1) Provided IR stretching frequencies isolated anthraquinone derivatives is not clear. All the derivatives with hydroxyl group but authors not mentioned the stretching frequencies of in data.

***Response: The IR stretching frequencies data of all 8 anthraquinone compounds has been published in Zamakshshari et al., 2017 – Cytotoxic activities of anthraquinones from morinda citrifolia towards SNU-1 and LS-174-T and K562 cell lines. Since this work is a continuation from Zamakshshari et al., 2017, we would think that there is no need to provide it again.***

2) Include 1H NMR and 13C NMR data of active molecule Morindone in the manuscript.

***Response: The data is provided in the supporting information file named S9_Table.pdf. This change can be tracked in the Supporting Information section in the revised manuscript.***

3) Include in vivo assay results.

***Response: We are looking forward to test this morindone using animal model. However, due to the limitation of the current funding, we are not able to test the morindone using animal model. Therefore, the in vivo work will be our next step, once we receive the upcoming funding for the study.***

After all these modification paper will be considered.

I am suggesting major revision.
