# Supplementary material for: Morindone from Morinda citrifolia as a potential antiproliferative agent against colorectal cancer cell lines
Source: PLoS One. 2022 Jul 12;17(7):e0270970. doi: 10.1371/journal.pone.0270970 (PMC9275698; doi:10.1371/journal.pone.0270970)
Supplement: Attachment — Submitted filename: Plos one revision.docx [file pone.0270970.s005.docx]

**Review comments**

Manuscript Number: PONE-D-21-32928

In this study, the authors extracted the potential anthraquinone compounds from the root bark of Morinda citrifolia. Eight potential anthraquinone compounds were successfully isolated, purified and tested for their anticancer efficacy.

Despite the fact that this work is a repetition of previously published work, authors must provide characterization data as well as scanned images of the active molecule.

I have suggested that, provided IR stretching frequencies some of the functional groups are missing, provide an active molecule scanned IR spectra.

The authors provided data in the table, but I suggest you to include scanned images of H^1^ NMR and C^13^ NMR of active molecule.

After all these modifications, the paper will be considered.

I am suggesting a major revision.
