# Supplementary material for: Morindone from Morinda citrifolia as a potential antiproliferative agent against colorectal cancer cell lines
Source: PLoS One. 2022 Jul 12;17(7):e0270970. doi: 10.1371/journal.pone.0270970 (PMC9275698; doi:10.1371/journal.pone.0270970)
Supplement: Attachment — Submitted filename: Response to reviewers 2.docx [file pone.0270970.s006.docx]

**Response to reviewers**

**Title: *Morindone from morinda citrifolia as a potential antiproliferative agent against colorectal cancer cell lines***

**Reference number: PONE-D-21-32928**

**Reviewer #2:** In this study, the authors extracted the potential anthraquinone compounds from the root bark of Morinda citrifolia. Eight potential anthraquinone compounds were successfully isolated, purified and tested for their anticancer efficacy.

Despite the fact that this work is a repetition of previously published work, authors must provide characterization data as well as scanned images of the active molecule.

Comment #1.

I have suggested that, provided IR stretching frequencies some of the functional groups are missing, provide an active molecule scanned IR spectra.

***Response: Thank you very much on your suggestion. We have now provided the active molecule (morindone) IR spectra under figure 8A of the results section.***

Comment #2.

The authors provided data in the table, but I suggest you to include scanned images of H1 NMR and C13 NMR of active molecule.

***Response: Thank you for the comment. We have now included the scanned images of H1 NMR and C13 NMR of the active molecule (morindone) under figure 8C and 8D, respectively.***

Comment #3.

After all these modifications, the paper will be considered.

***Response: Thank you very much for your valuable comments and they certainly improved our paper for publication.***
